# Accumulation of CD103^+^ CD8^+^ T cells in a cutaneous melanoma micrometastasis

**DOI:** 10.1002/cti2.1100

**Published:** 2019-12-25

**Authors:** Katharina Hochheiser, Han Xian Aw Yeang, Teagan Wagner, Candani Tutuka, Andreas Behren, Jason Waithman, Christopher Angel, Paul J Neeson, Thomas Gebhardt, David E Gyorki

**Affiliations:** ^1^ Peter MacCallum Cancer Centre Melbourne VIC Australia; ^2^ Department of Microbiology & Immunology The University of Melbourne at the Peter Doherty Institute for Infection & Immunity Melbourne VIC Australia; ^3^ Sir Peter MacCallum Department of Oncology The University of Melbourne Parkville VIC Australia; ^4^ Telethon Kids Institute University of Western Australia Perth WA Australia; ^5^ Olivia Newton‐John Cancer Research Institute Heidelberg VIC Australia; ^6^ Department of Surgery University of Melbourne Melbourne VIC Australia

**Keywords:** melanoma, micrometastasis, tissue‐resident memory T cells

## Abstract

**Objective:**

The immune system can halt cancer progression by suppressing outgrowth of clinically occult micrometastases in a state of cancer‐immune equilibrium. Cutaneous melanoma provides a unique opportunity to study the immune contexture of such lesions, as miniscule skin metastases are accessible to clinical inspection and diagnostic biopsy.

**Methods:**

Here, we analysed by multiplex immunofluorescence microscopy samples from a melanoma patient presenting with an overt and an occult in‐transit metastasis (ITM), the latter of which appeared as a small erythematous papule.

**Results:**

Microarchitecture and immune composition in the two lesions were vastly different. CD4^+^ and CD8^+^ T cells accumulated around the margin of the overt SOX10^+^ Melan A^+^ ITM but were largely excluded from the tumor centre. By contrast, the occult micrometastasis contained only few SOX10^+^ Melan A^−^ melanoma cells which were scattered within a dense infiltrate of T cells, including a prominent population of CD103^+^ CD8^+^ T cells resembling tissue‐resident memory T (T_RM_) cells. Notably, almost every single melanoma cell in the micrometastasis was in close proximity to these T_RM_‐like cells.

**Conclusion:**

Such results support the emerging concept that CD103^+^ CD8^+^ T_RM_ cells are key mediators of cancer surveillance and imply an important function of these cells in controlling clinically occult micrometastases in humans.

## Graphical abstract

Analysing an occult in‐transit metastasis in a melanoma patient, we identify CD103^+^CD8^+^ T cells with a resident memory phenotype as the dominant T‐cell subset. Such results support the emerging concept that CD103^+^CD8^+^ tissue‐resident memory T cells are key mediators of cancer surveillance and imply an important function of these cells in controlling clinically occult micrometastases in humans.

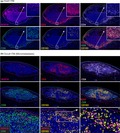

## Introduction

Most patients diagnosed with cutaneous melanoma undergo surgical excision of the primary tumor and never develop overt metastatic disease. For those patients whose melanoma recurs, the anatomical and temporal pattern is highly variable. Approximately 5% of melanoma patients present with in‐transit metastases (ITM) which represent locoregional intralymphatic recurrences in the dermal or subdermal lymphatics between the site of the primary tumor and the draining lymph nodes.^1^ The median time from primary melanoma surgery to presentation with ITM is 17–18 months, which indicates a certain degree of control of metastasising cancer cells, most likely mediated by the immune system. In fact, it is becoming increasingly apparent that the immune system plays an important role in eradicating cancer cells or keeping cancer cells that resist such eradication in a dormant state.^2^, ^3^, ^4^, ^5^ The latter mode of control is often referred to as cancer‐immune equilibrium and can be maintained for several decades,^2^, ^4^, ^5^, ^6^ whereas recurrence most likely reflects a loss of immune control. The immune composition of controlled micrometastases that are maintained in cancer‐immune equilibrium, however, has remained inherently difficult to determine because of their often clinically inapparent nature.

The population of tumor‐associated lymphocytes in many advanced cancers contains CD8^+^ T cells that express transcriptional hallmarks of tissue‐resident memory T (T_RM_) cells, including a lack of expression of the transcription factor, Kruppel‐like factor 2 and, consequently, an absence of tissue‐exit receptors such as S1PR1 and CCR7.^7^, ^8^, ^9^ T_RM_ cells are sessile cells that permanently reside within tissues without recirculating around the body.^10^ In the epidermal layer of skin and other epithelial tissues, T_RM_ cells commonly express surface markers such as CD69 and CD103 which discriminate them from recirculating memory T cells.^11^ Importantly, T_RM_ cells in skin are key mediators of localised protection from infection with viruses and other pathogens.^10^ However, T_RM_ cells can also drive skin inflammation in the context of autoimmune diseases and tissue transplantation.^12^ Emerging clinical data further suggest that T_RM_ cells provide immune surveillance in a variety of human cancers.^10^, ^13^ Importantly, the density of T_RM_ cells within the tumor environment has been shown to predict for improved survival in a number of cancer types,^7^, ^9^, ^10^, ^13^, ^14^ including advanced‐stage melanoma.^15^ In line with this, recent reports have shown that T_RM_ cells afford potent antitumor immunity in mouse models.^10^, ^13^, ^16^, ^17^, ^18^ Furthermore, we have recently demonstrated that T_RM_ cells can promote cancer‐immune equilibrium in mouse skin by keeping dispersed melanoma cells dormant over extended periods of time.^19^ Whether T_RM_ cells have a similar function in maintaining tumor dormancy in humans, however, remains to be addressed. Of note in this regard, previous clinical studies have focused on T_RM_ accumulation in large tumors, in which immune control has most likely failed. Thus, the role of T_RM_ cells in mediating cancer‐immune equilibrium of dormant cancer cells and micrometastases remains unresolved.

## The clinical case

**Figure 1:**
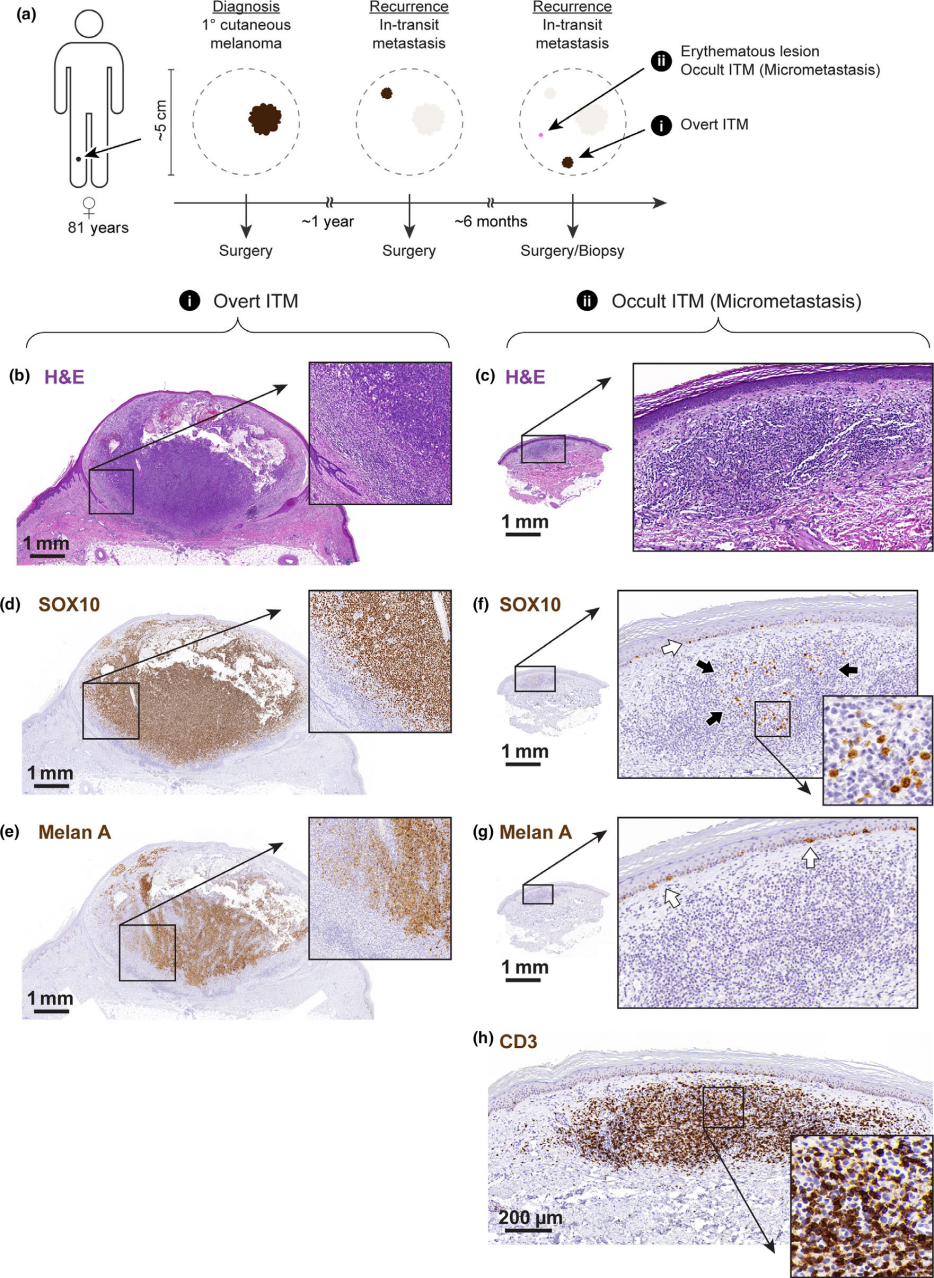
Cartoon depicting the time line and anatomical distribution of the analysed lesions **(a)**. Histopathological analysis of the overt in‐transit metastasis and micrometastasis using haematoxylin and eosin **(b, c)**, anti‐SOX10, **(d, f)** anti‐Melan A **(e, g)** and anti‐CD3 **(h)** staining. Higher magnification photographs correspond to regions of interest, as indicated. Black arrows in **(f)** indicate scattered SOX10^+^ melanoma cells; white arrows in **(f)** and **(g)** indicate SOX10^+^ Melan A^+^ melanocytes in the epidermal layer of skin.

An 82‐year‐old woman presented with recurrent dermal melanoma ITMs (Figure 1a). Two years prior to presentation, she underwent a wide excision for a 1.5‐mm Breslow thickness (T2) cutaneous melanoma on the right lower leg. Twelve months later, the patient developed an isolated dermal in‐transit recurrence ~ 4 cm from the primary site which was excised. She did not receive any adjuvant systemic therapy. A further 7 months later, a new pigmented dermal ITM of ~ 6 mm in diameter was identified on the lower leg. The patient also identified an erythematous lesion on the same leg, ~ 2 mm in diameter, which she described as resembling the precursors to the two overt ITMs.

Both lesions were excised and examined histologically. Haematoxylin and eosin staining of sections of the overt ITM revealed a nodular tumor in the superficial dermis with a diameter of ~ 6 mm and a thickness of ~ 3.5 mm and multiple areas of cystic degeneration. Around the periphery of the tumor was a moderate lymphocytic infiltrate (Figure 1b). By contrast, the erythematous papule corresponded to a lymphocytic infiltrate in the papillary dermis and had a diameter of ~ 2 mm and a thickness of ~ 0.4 mm (Figure 1c). Immunohistochemical staining confirmed that the overt ITM composed of melanoma cells that uniformly stained positive for SOX10 (Figure 1d). The staining pattern for melanocyte antigen A (Melan A; or melanocyte antigen recognised by T cells 1) was more heterogeneous, with notable patches of Melan A^−^ cells in the tumor centre and in close proximity to the lymphocytic infiltrate at the tumor margin (Figure 1e). The occult erythematous lesion contained only a low number of SOX10^+^ melanoma cells (Figure 1f) and these uniformly lacked expression of Melan A (Figure 1g). Notably, these SOX10^+^ melanoma cells were embedded within a dense dermal infiltrate of CD3^+^ T cells that made up the overall volume of this lesion (Figure 1h). Nevertheless, the presence of SOX10^+^ melanoma cells identified this lesion as an occult, potentially immune controlled, regressed or recently escaped ITM.

Six months following the surgery for the analysed lesions, the patient underwent repeat PET scan and was found to have multiple new ITMs in addition to a new metastasis in an inguinal lymph node. She was started on single‐agent pembrolizumab (anti‐PD1) treatment and achieved a rapid complete response after 3 months of treatment. Nine months after starting checkpoint blockade immunotherapy, the patient continues to have a complete response.

## Results

**Figure 2:**
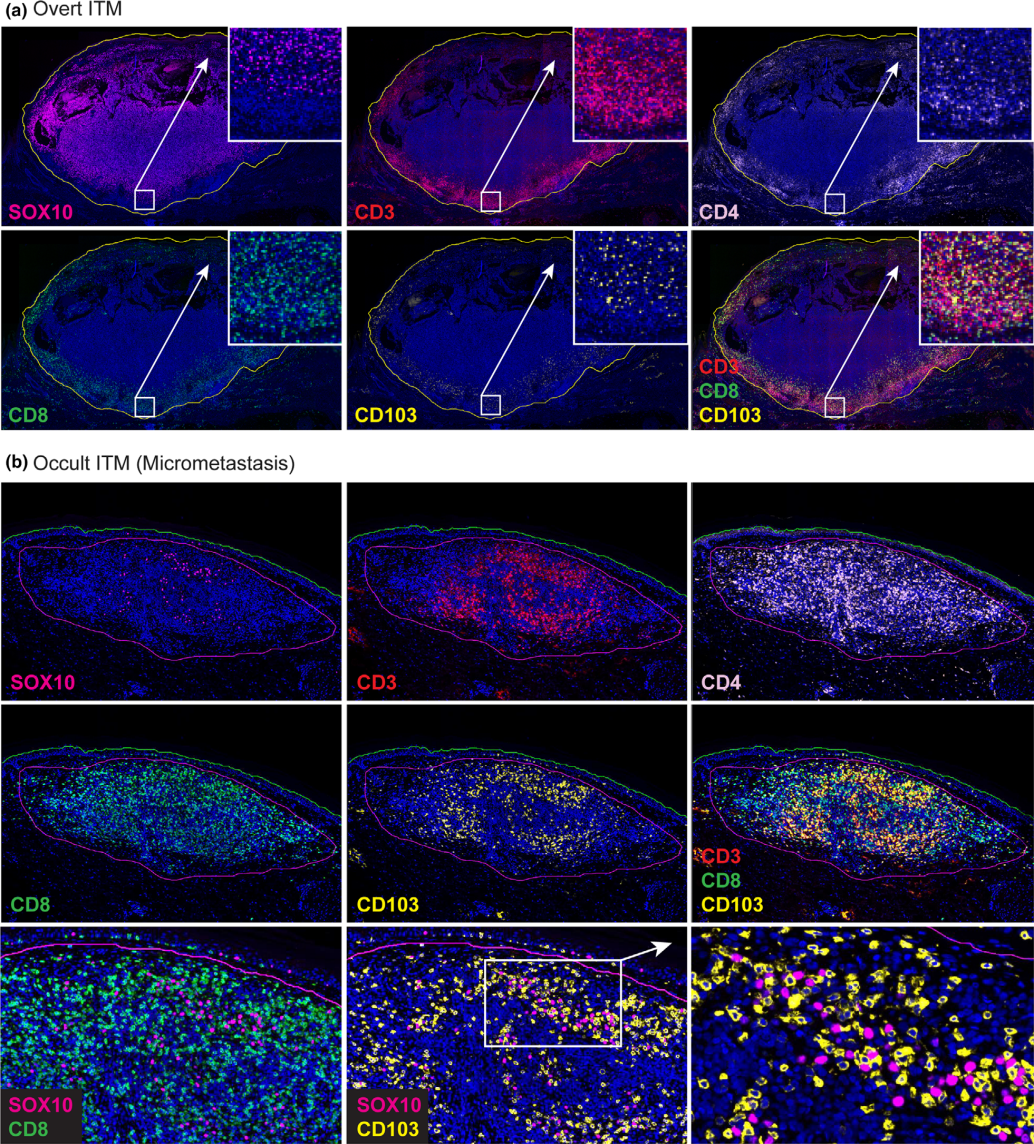
Multiplex immunofluorescence analysis of the overt **(a)** and the occult in‐transit metastasis (micrometastasis) **(b)** using antibodies against SOX10, CD3, CD4, CD8 and CD103 (colours indicated in overlay text). Additional FOXP3 staining not shown. Cell nuclei counterstained with DAPI (blue).

**Figure 3:**
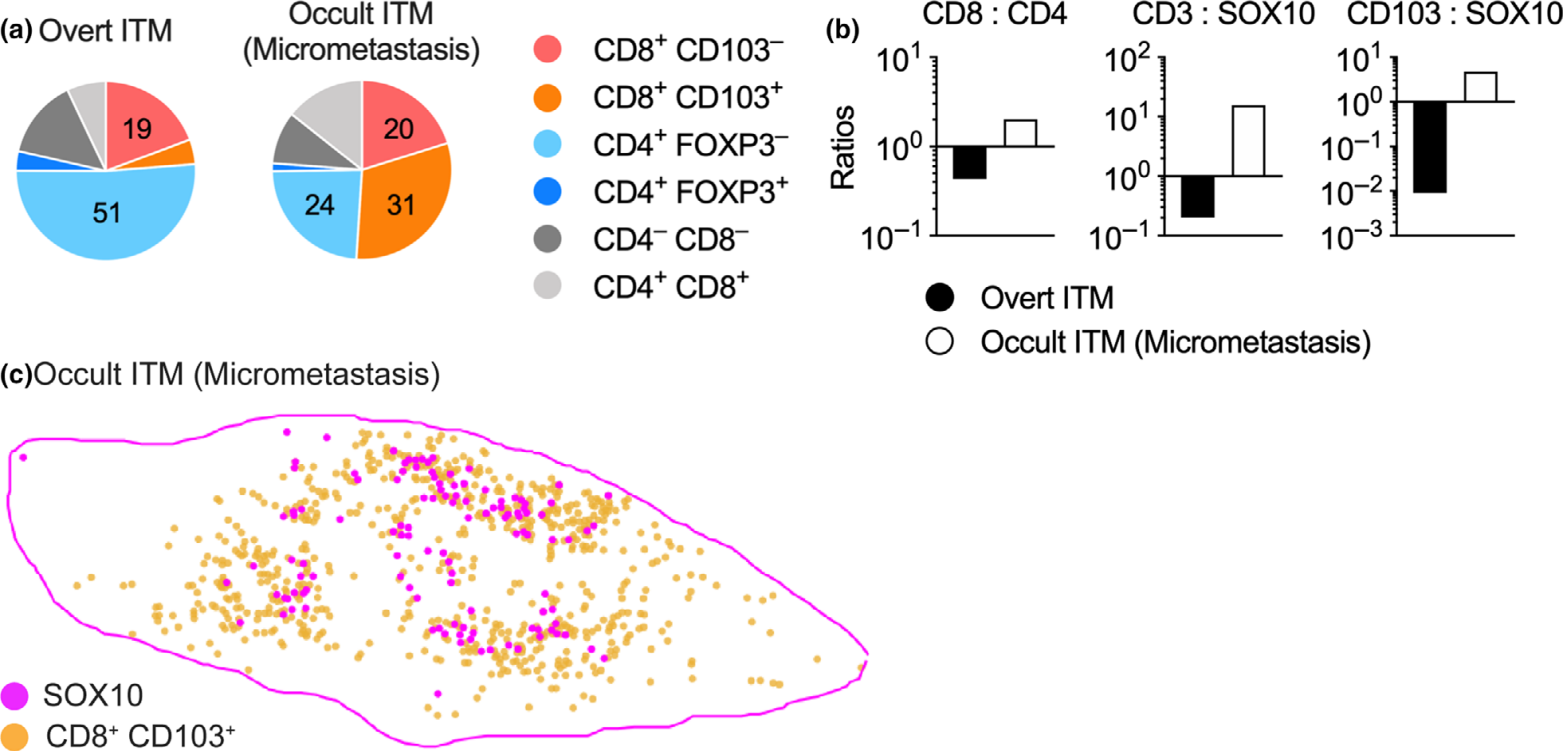
Relative distribution (percentage, **a**) and ratios **(b)** of T‐cell subsets in the overt in‐transit metastasis and the occult micrometastasis as calculated from the analysis in Figure 2. **(c)** Spatial distribution of CD103^+^ CD8^+^ T_RM_‐like cells (orange) relative to SOX10^+^ melanoma (magenta) cells in the micrometastasis, *n* = 1.

This case offered a unique opportunity to compare the immune contexture of an overt ITM and a clinically occult micrometastasis within the same patient. Thus, we performed multiplex immunohistochemistry (IHC) on these samples, employing an antibody panel that allowed us to visualise the spatial distribution of melanoma cells relative to CD3^+^ T‐cell subsets, including CD8^+^ CD103^+^ T_RM_ cells. The overt ITM consisted of a solid centre of SOX10^+^ melanoma cells which was only sparsely infiltrated by T cells. Instead, CD3^+^ T cells formed a noticeable band‐like infiltrate at the tumor margin (Figure 2a). This infiltrate was dominated by CD4^+^ T cells (54.8% of CD3^+^ cells; Figure 3a and b) but also composed of CD8^+^ T cells (23.8% of CD3^+^ cells), some of which co‐expressed the T_RM_ marker CD103 (18.9% of all CD8^+^ T cells, Figures 2a and 3a). Approximately, 3.5% of all T cells and 6.5% of CD4^+^ T cells in this lesion were CD4^+^ regulatory T (T_REG_) cells, as indicated by their expression of the transcription factor FOXP3 (Figure 3a and not shown). The occult micrometastasis contained scattered, isolated SOX‐10^+^ melanoma cells which were surrounded by CD3^+^ T cells and were thus in immediate proximity to CD4^+^ as well as CD8^+^ T cells (Figure 2b). In contrast to the overt ITM, the majority of T cells in the occult lesion were CD8^+^ (51% of CD3^+^ cells, Figure 3a and b), including a dominant population of CD8^+^ CD103^+^ T cells (Figures 2b and 3a). These T_RM_‐phenotype cells made up 30.9% of all T cells and 60.5% of CD8^+^ CD3^+^ T cells (Figure 3a), whereas FOXP3^+^ CD4^+^ T_REG_ cells were sparse and made up <1.4% of all CD3^+^ T cells and 5.7% of CD4^+^ T cells (Figure 3a). Of note, approximately a quarter of CD3^+^ T cells in the two lesions either lacked expression of CD4 and CD8 markers or, alternatively, expressed both simultaneously, and therefore may represent unconventional T cells (Figure 3a).

In summary, the two lesions displayed a vastly dissimilar microarchitecture with marked differences in the abundance of T‐cell subsets and their spatial distribution relative to melanoma cells. Consequently, most SOX10^+^ cells in the overt ITM were in considerable distance to T cells, while in the occult micrometastasis virtually all SOX10^+^ cells were surrounded by and in direct contact with T cells (Figure 2b). This was echoed by a considerably higher overall ratio of CD3^+^ T cells to SOX10^+^ melanoma cells in the occult micrometastasis which was 15.7 compared to 0.2 for the overt ITM (Figure 3b). Likewise, the ratio of CD103^+^ CD8^+^ T cells to SOX10^+^ cells was 4.86 for the occult micrometastasis and 0.009 for the overt ITM, respectively (Figure 3b). Notably, almost every single melanoma cell in the micrometastasis was found in close proximity to CD103^+^ CD8^+^ T cells (Figures 2b and 3c). By contrast, CD103^+^ CD8^+^ T cells in the same sections did not accumulate in proximity to SOX10‐expressing normal melanocytes located in the epidermal layer of skin (Figure 2b). Thus, the micrometastasis displayed a T‐cell‐rich microenvironment with T_RM_‐like cells representing the predominant T‐cell subset.

## Discussion

For most patients with metastatic cancer, the early stages of metastasis are difficult to identify and measure given the limitations of imaging techniques. Dermal metastases from cutaneous melanoma are readily visible to the naked eye and can be identified from a very small size. This case report describes what we believe to be the closest situation to immune equilibrium or potentially the earliest phase of escape from immune control of a tumor metastasis.

A number of recent preclinical studies have revealed an important role for T_RM_ cells in melanoma surveillance. In line with this, we have recently shown that CD8^+^ CD103^+^ T_RM_ cells promote cancer‐immune equilibrium and thereby prevent the outgrowth of residual melanoma cells that can persist in mouse skin over extended periods of time.^19^ Accordingly, the generation of large numbers of T_RM_ cells in skin is associated with improved immune control and suppressed cancer progression. Several recent reports in a multitude of human cancers, including melanoma, suggest that T_RM_ cells can promote immune surveillance and that their strong accumulation serves as a prognostic biomarker for improved clinical outcomes.^7^, ^9^, ^14^, ^15^ However, those studies have focused on advanced and, most likely, immune‐escaped tumors. Thus, whether T_RM_ cells are able to drive cancer‐immune equilibrium and prevent progression of dormant cancer cells in humans has remained unaddressed.

This case of an 82‐year‐old patient, who presented with an overt melanoma ITM in the left calf and an adjacent erythematous lesion that appeared to be a potentially dormant micrometastasis, offered an unique opportunity to compare in the same individual the immune environments associated with immune escape (overt ITM) and cancer‐immune equilibrium, or the transition to early escape or regression, respectively (occult ITM/micrometastasis). In line with vastly different immune environments, the melanoma cells in both lesions expressed SOX10, but those in the micrometastasis uniformly lacked expression of the melanocyte lineage‐specific marker, Melan A. The lack of Melan A expression could reflect genetic selection for Melan A^−^ escape variants under immune pressure or alternatively, a degree of reversible dedifferentiation of the melanoma cells in the micrometastasis. The latter represents an immune resistance mechanism driven by the proinflammatory cytokine TNFα and likely at play in this T‐cell‐rich lesion.^20^, ^21^ In support of this notion, patches of Melan A^−^ melanoma cells in the overt ITM were found in direct vicinity to the T‐cell infiltrate at the tumor margin, whereas the majority of melanoma cells within the tumor centre and in distance to T cells retained expression of Melan A. It should be noted that SOX10 expression in skin is not restricted to cells of the melanocytic lineage, but can also be detected in cells of neural and myoepithelial origin and in histiocytes, the latter of which belong to the macrophage lineage and accumulate in scar tissue.^22^ Thus, we cannot formally exclude the possibility that the Melan A^−^ SOX10^+^ cells in the occult lesion may represent cells other than melanoma cells. However, as the patient described the erythematous lesion to resemble the precursors of the previous escaped ITMs, and given that it was distant to the original excision site and did not appear histologically as scar tissue, those cells are likely to represent melanoma cells under immune pressure.

In the occult micrometastasis, we observed a prominent population of CD8^+^ CD103^+^ T cells in close proximity to SOX10^+^ melanoma cells, suggesting that human T_RM_ cells can survey dormant melanoma cells and maintain immune equilibrium. This is in line with recent reports demonstrating the presence of CD103^+^ T_RM_ in close proximity to tumor cells in patients with different types of cancers, including melanoma and breast cancer.^9^, ^15^ Such results further echo our findings in mouse skin where T_RM_ cells can directly interact with and control dormant melanoma microlesions.^19^ Furthermore, clustering of antigen‐specific T_RM_ cells with infected cells has been observed in the context of genital Herpes simplex virus (HSV)‐2 infection in human genital skin and in response to HSV‐1‐infection in mouse skin, suggesting that accumulation of T_RM_ cells preferentially occurs in the context of abnormal or infected but not normal cells.^23^, ^24^ In line with this, CD103^+^ CD8^+^ T cells did not appear to cluster with SOX10‐expressing normal melanocytes in the epidermis, indicating that the accumulation of T_RM_‐like cells around SOX‐10^+^ cells was because of the abnormal nature of those cells. T_RM_ cells were comparatively sparse in the overt ITM and were, together with other T‐cell subsets, largely excluded from the centre and restricted to the tumor margin. This again reflects a cohort of escaped tumors in preclinical models, where T_RM_ cells are mainly found at the margin but not in the centre of progressing tumors.^19^

It should be noted, however, that there are a few inherent limitations in interpreting our results. Firstly, this is a single case. Even though the data obtained from those lesions go hand in hand with what has previously been described in mouse models, at this stage, we cannot exclude that those similarities are coincidental or at least not representative. Secondly, the two biopsies only represent a snapshot of what the metastases and the immune environments looked like at the time of surgery. Clearly, the overt metastasis had escaped immune surveillance and was progressively growing before the surgery. However, the natural history of the occult metastasis is unpredictable. It may represent the first step in escape from immune equilibrium, or, as seen in mouse models, it may also reflect temporary tumor growth before return to immune control and quiescence,^19^ which could have lasted for a prolonged period of time if not indefinitely. Lastly, although tumor‐associated CD103^+^ T_RM_‐like cells are often enriched for cancer‐specific cells,^25^ a large fraction of these may be made up of virus‐specific T_RM_ cells that were generated during previous infections.^26^, ^27^ As such, these bystander cells would be unable to recognise tumor antigens, meaning that a proportion of the CD103^+^ T_RM_‐like cells may not actively take part in antitumor immunity. Regardless, in conjunction with recent clinical and preclinical studies, this case strongly implies a role for T_RM_ cells in providing cancer‐immune surveillance and promoting cancer‐immune equilibrium in human in‐transit melanoma metastases and highlights the potential of targeting T_RM_ for future cancer immunotherapies.

## Methods

### Haematoxylin and eosin and immunohistochemistry

Tissues were fixed in formalin and embedded in paraffin. Three‐micrometer sections were stained with haematoxylin and eosin (H&E) or subjected to IHC in the Ventana automated slide stainer (Ventana Medical Systems, Oro Valley, AZ, USA), using the OptiView DAB IHC detection kit (Roche, Basel, Switzerland) according to the manufacturer's instructions. The following antibodies and dilutions were used: anti‐human CD3 clone SP7 (Spring Bioscience, Pleasanton, CA, USA) used at 1:100; anti‐human SOX10 clone BC34 (Biocare Medical, Pacheco, CA, USA) used at 1:50; and anti‐Melan A clone A103 (Novocastra, Newcastle, UK) used at 1:50. Slides were imaged on the Pannoramic SCAN II Digital Slide Scanner (3DHistech, Budapest, Hungary).

### Multiplex immunohistochemistry (mIHC; Opal™) staining, imaging and data analysis

Multiplex IHC was performed as per our previous study with minor modifications.^28^ Briefly, 3‐µm‐thick sections of formalin‐fixed, paraffin‐embedded tissue were cut onto Trajan Series 3 slides (Trajan Sci Med, Ringwood, VIC, Australia). Slides were then loaded onto a BOND RX fully automated research stainer (Leica Biosystems, Mount Waverley, VIC, Australia), and an optimised serial protocol consisting of dewaxing, heat‐induced epitope retrieval, blocking with hydrogen peroxide, staining with primary antibodies and Opal polymer HRP, Opal TSA amplification, washing steps and DAPI counterstain was performed (Table 1).

All slides were imaged on a Vectra® three automated quantitative pathology imaging system (PerkinElmer, Waltham, MA, USA) at 20×. Multispectral images were deconvoluted within inForm® software (PerkinElmer) using multispectral library reference slides that consist of single‐colour slides for each of the six Opal fluorophores and one DAPI stain.

Multiple individual deconvoluted images for each lesion were stitched together in the HALO™ image analysis platform (Indica Labs, Albuquerque, NM, USA). Tissue region was manually annotated, and thresholds for individual fluorophore channel were determined for each tissue using the High‐Plex FL module. Spatial plots were generated, and cell density was calculated within HALO™. Individual cell statistics were exported for further analysis in GraphPad Prism 7 (GraphPad Software, San Diego, CA, USA).

**Table 1:** Reagents used for mIHC

Reagent	Dilution	Clone	Manufacturer
CD4	1:100	SP35	Spring Bioscience
CD8	1:1000	4B11	Thermo Fisher Scientific Inc, Waltham, MA, USA
SOX10	1:200	BC34	Biocare Medical
FOXP3	1:100	Polyclonal	BioSB, Nijmegen, Netherlands
CD103	1:2000	EPR4166(2)	Abcam, Cambridge, UK
CD3	1:1000	SP7	Spring Bioscience
Opal Polymer HRP	1:1	–	PerkinElmer
DAPI	1 drop mL^−1^	–	PerkinElmer
Opal 7‐colour IHC Kit	–	–	PerkinElmer

## Conflict of interest

The authors declare no conflict of interest.
